# Static Visual Agnosia Following Awake Resection of a Left Frontal Low-Grade Glioma: A Case Report of Ventral Stream Network Disruption (“Astatopsia”)

**DOI:** 10.3390/reports9010001

**Published:** 2025-12-19

**Authors:** Stefano Vecchioni, Alessio Iacoangeli, Andrea De Angelis, Silvia Bonifazi, Roberto Trignani, Michele Luzi

**Affiliations:** 1 Department of Neurosurgery, Azienda Ospedaliero Universitaria delle Marche, 60126 Ancona, Italy; stefano.vecchioni@ospedaliriuniti.marche.it (S.V.); dandrea341@yahoo.com (A.D.A.); roberto.trignani@ospedaliriuniti.marche.it (R.T.); michele.luzi@ospedaliriuniti.marche.it (M.L.); 2 Department of Neurosurgery, Ospedale IRCSS San Martino, Università degli Studi di Genova, 16132 Genova, Italy; 3 Department of Clinical Health Psychology, Azienda Ospedaliero Universitaria delle Marche, 60126 Ancona, Italy; silvia.bonifazi@ospedaliriuniti.marche.it

**Keywords:** astatopsia, visual agnosia, frontal glioma, dynamic aphasia, dysexecutive syndrome, motion perception, dual-stream model, awake brain surgery, static image recognition, hodotopy

## Abstract

**Background and Clinical Significance**: Visual agnosia and speech production deficits are well-described sequelae of neurosurgical interventions, but their selective dissociation remains rare. This report presents an unusual combination of postoperative deficits following awake resection of a left frontal low-grade glioma. **Case Presentation**: We present the case of a right-handed female with left hemisphere language dominance who had a left frontal low-grade glioma. Preoperatively, she exhibited anomia and dysexecutive syndrome, including difficulty completing everyday goal-directed tasks such as sending emails and paying for parking. Following awake tumor resection, she developed two rare, dissociated deficits: (1) speech restricted to infinitive verb forms and (2) selective visual agnosia for static images, with preserved recognition of dynamic stimuli. **Conclusions**: This uncommon clinical constellation highlights the vulnerability of left frontal language and ventral visual processing networks during surgery and supports the dual-stream model of vision and language production. We describe a selective form of static visual agnosia affecting static images with relative preservation of dynamic and object recognition, for which we use the descriptive label “astatopsia”. This peculiar clinical condition is rarely documented in this particular combination and has not, to the best of our knowledge, previously been denominated in such a manner in the literature.

## 1. Introduction and Clinical Significance

In most right-handed individuals, language functions are predominantly supported by the left hemisphere [1]. Low-grade gliomas in the left frontal lobe can therefore affect both language and executive functions, and awake surgery with intraoperative mapping is commonly used to minimize postoperative deficits. Visual agnosias—impairments in object recognition despite relatively well-preserved basic visual functions—classically arise from occipitotemporal damage and are broadly classified as apperceptive, associative, or integrative agnosias. Dissociations between perception of static and dynamic visual stimuli have been documented in several contexts, including Riddoch’s early description of patients with occipital lesions who reported perception confined to movement in an otherwise blind field, and in variants of blindsight where motion or location can be processed in the absence of conscious form perception [2]. More recently, the dual-stream model of visual processing has distinguished a ventral “what” pathway supporting object recognition from a dorsal “wherehow” pathway supporting spatial vision and visually guided action [3,4]. Within this framework, motion processing in areas such as hMT/V5 can be supported by alternative geniculo-extrastriate routes, as demonstrated in blindsight [5]. Dissociations between static form and motion perception have also been described in integrative visual agnosia and related conditions [5,6,7], as well as in classic static visual agnosias (e.g., Botez, 1967) [8,9].

In this article, we describe the case of a patient who developed a selective postoperative difficulty recognizing static images in the presence of relatively well-preserved recognition of moving stimuli and real objects, together with a dynamic aphasia-like speech production disturbance (infinitive-only speech), after awake resection of a left frontal low-grade glioma. We coined the term “astatopsia” descriptively to refer to this pattern of impaired static image recognition, which can be situated within the broader category of static visual agnosias. The detailed case documentation, including formal pre- and postoperative neuropsychological data, provides insight into the interaction between frontal language networks and ventral visual processing pathways.

## 2. Case Presentation

### 2.1. Patient Presentation Summary

A 52-year-old native Italian-speaking right-handed woman was referred for evaluation of a left frontal lesion adjacent to Broca’s area. Handedness was assessed with the Edinburgh Handedness Inventory [10], and language lateralization was further explored with Kimura’s dichotic listening test [11], both of which strongly suggested left-hemisphere dominance for language. There was no prior neurological or psychiatric history.

Preoperatively, she reported gradual onset of word-finding difficulties and problems with everyday goal-directed behavior. Clinical and neuropsychological evaluation revealed:

Anomia: difficulty naming objects despite generally fluent conversational speech.

Dysexecutive syndrome: difficulty planning and executing complex tasks, such as organizing emails and completing payment procedures (e.g., paying for parking), consistent with dysfunction involving the left dorsolateral prefrontal cortex [3].

Motor, sensory, and visual fields: intact on neurological examination.

Basic visual functions: no deficit in acuity, contrast sensitivity, and visual fields testing.

A comprehensive neuropsychological battery was administered, including measures of language, attention, memory, executive functions, praxis, visuospatial skills, and gnosia, according to the evaluation protocol used in our center: naming test, visual object recognition, semantic tests, etc. The results are summarized in Section 2.7 and Table 1.

Briefly, preoperative testing showed mild anomia on visual naming, impaired performance on executive function tests, and preserved visuospatial skills and visual object recognition. Two new dissociated deficits emerged post-surgery: output restricted to infinitive verb forms and selective visual agnosia for static images (but dynamic recognition preserved). The overall condition was called “astatopsia”.

### 2.2. Preoperative Neuroimaging

**Figure 1:**
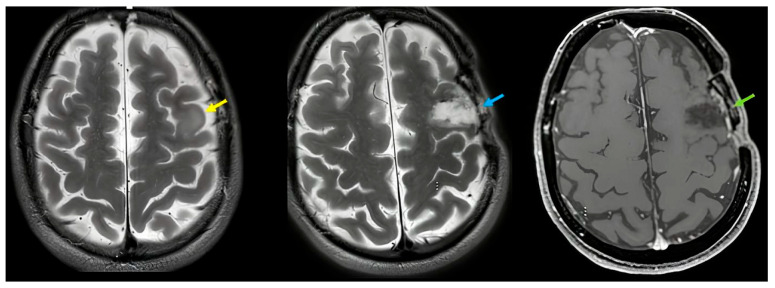
(**Left image**) Area of signal increase in T2/FLAIR affecting the middle frontal lobe at the left convexity, with a slightly swollen appearance, with blurred and irregular margins, and with partial involvement of the cortex. This alteration shows no signs of diffusion restriction, does not appear hyperperfused, and does not show signs of an altered blood–brain barrier after IV administration of the contrast agent. In the spectroscopic study conducted at this level, a slight reduction in the NAA peak is observed (yellow arrow). (**Central image**) Postoperative T2 image demonstrating gross total resection (blue arrow). (**Right image**) Postoperative T1 image demonstrating gross total resection (green arrow).

Preoperative MRI demonstrated a low-grade glioma involving the left inferior and middle frontal gyri, with partial involvement of cortical and subcortical white matter adjacent to Broca’s area (Figure 1). The lesion appeared hyperintense on T2/FLAIR images, without diffusion restriction or contrast enhancement. Spectroscopy showed a slight reduction in the NAA peak, compatible with a low-grade infiltrative glioma.

### 2.3. Surgical Procedure and Intraoperative Mapping

#### 2.3.1. Overview

The patient underwent awake craniotomy with cortical and subcortical mapping. After dural opening, classical Penfield bipolar stimulation and a four-channel cortical strip electrode were used to identify language and executive function sites.

The low-grade glioma was centered in the left inferior frontal gyrus (IFG) with superior extension into the middle frontal gyrus (MFG), abutting the posterior IFG near the precentral sulcus.

**Figure 2:**
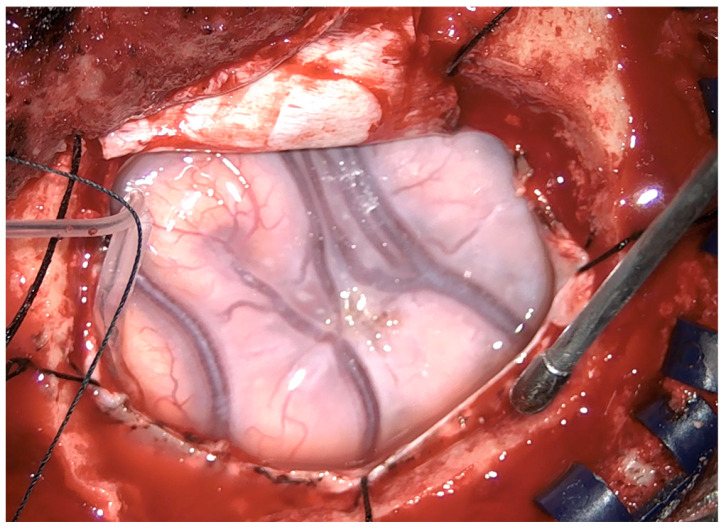
Intraoperative finding: swollen appearance of the frontal lobe and bluish aspect of the glioma.

Intraoperative findings revealed a bluish, swollen appearance of the frontal lobe lesion, consistent with low-grade glioma (Figure 2). Gross total resection of the tumor was achieved under continuous clinical monitoring.

#### 2.3.2. Surgical Details

A total awake protocol was used to enable direct electrical stimulation (DES) mapping while maximizing extent of resection (EOR) within the language-eloquent frontal cortex. Intraoperatively, cortical DES delineated a posterior IFG “no-go” zone: stimulation over the pars opercularis/ventral precentral region reproducibly induced speech arrest and articulatory breakdown, while stimulation over the pars triangularis induced morphosyntactic disruption during sentence completion (responses collapsed to uninflected/infinitive-like verb forms despite preserved lexical access). Resection was therefore pursued through a sulcus-based transcortical corridor in the anterior MFG and anterior IFG—entering anterior to the mapped posterior IFG language sites, proceeding posteriorly until functional boundaries were reached, and continuing in a subpial fashion inferiorly along the inferior frontal sulcus while sparing the posterior opercular cortex. Subcortical DES at the posterior–superior depth of the cavity elicited arrest/hesitation and “speech initiation failure” consistent with stimulation of frontal language outflow pathways, leading to termination of resection at that margin. At the antero-inferior depth, DES triggered selective failures in static object recognition (inability to identify photographs/line drawings or to match objects to semantic categories), while recognition of brief dynamic stimuli (short video clips depicting the same objects/actions) remained preserved, prompting the surgeon to stop inferior/deeper resection to avoid further disruption of ventral semantic access pathways.

Postoperatively, the patient presented with (1) productive language restricted largely to infinitive verb forms, most evident in constrained sentence completion and spontaneous speech where tense/agreement marking was absent despite intact comprehension and noun retrieval, closely mirroring the intraoperative morphosyntactic errors evoked by pars triangularis stimulation and consistent with functional disturbance of the posterior IFG–subcortical “grammar/selection” network along the posterior resection boundary; and (2) a selective visual agnosia for static images with preserved recognition of dynamic stimuli, aligning with the intraoperative finding that deep antero-inferior stimulation near the inferior frontal resection floor perturbed static-picture semantic access while sparing motion-driven recognition, suggesting partial disturbance (e.g., edema/traction or micro-disconnection) of the fronto-occipito-temporal semantic pathways encountered at the inferior/deeper limit of the surgical corridor. The final EOR was near-total/subtotal by functional criteria, with a deliberate residual left at the posterior inferior margin adjacent to the cortical “speech arrest” sites and the subcortical region where stimulation produced morphosyntactic and static visual recognition disturbances, explicitly linking the achieved resection boundaries to the pattern of postoperative deficits.

##### Intraoperative Mapping Protocol (Penfield Bipolar Technique)

Stimulation technique and parameters: Cortical and subcortical DES was performed using the classic Penfield bipolar method (bipolar probe with ~5 mm interelectrode distance) under continuous electrocorticography (ECoG) to detect afterdischarges. Biphasic square-wave stimulation was delivered at 60 Hz, pulse width ~1 ms per phase, in 2–4 s trains. Current intensity began at 2 mA and was increased stepwise (up to 6 mA on cortex; up to 7 mA subcortically) to the lowest intensity that produced a reproducible behavioral effect without afterdischarges. If afterdischarges occurred, stimulation was stopped, the cortex was irrigated with cold Ringer’s lactate, and mapping was resumed at a lower intensity.

##### Awake Task Battery with Real-Time Performance Monitoring

Tasks were selected to probe (i) speech output, (ii) morphosyntax/verb inflection, and (iii) visual–semantic recognition under both static and dynamic conditions:

Counting and automatic speech (baseline fluency/speech arrest screening);

Picture naming (static) using photographs/line drawings;

Verb generation (produce an action verb from a presented noun);

Sentence completion with inflection (e.g., prompts requiring tense/agreement marking rather than lemma retrieval);

Reading aloud and repetition (phonological output control);

Visual recognition: static vs. dynamic (matching static images to spoken labels; brief video clips of the same objects/actions to compare recognition routes);

Functionally positive cortical sites (representative intraoperative findings);

Posterior IFG/ventral precentral region (pars opercularis–ventral PrCG): stimulation caused speech arrest and/or severe articulatory disruption (task interruption during counting/naming), defining the posterior resection limit.

Pars triangularis (anterior-to-posterior IFG transition): stimulation caused morphosyntactic breakdown; during sentence completion, the patient produced uninflected/infinitive-like verb forms or default lemmas despite preserved comprehension and noun retrieval, marking a language-critical zone relevant to postoperative infinitive-only speech.

Anterior MFG: stimulation was functionally silent for language tasks (used as a safer entry region for the transcortical corridor);

Functionally positive subcortical sites (representative intraoperative findings);

Posterior–superior depth of the cavity: stimulation induced speech initiation failure/hesitation (and occasional negative motor-like arrest), prompting cessation of resection at that margin to protect frontal language outflow pathways;

Antero-inferior depth (inferior frontal floor): stimulation produced selective impairment on static object recognition/semantic matching, while dynamic stimulus recognition remained intact, leading to termination of inferior/deeper resection and providing an intraoperative analogue of the postoperative selective static visual agnosia.

##### Surgical Corridor and EOR

The operative corridor was planned to avoid the posterior IFG speech-arrest sites, adopting a transcortical/sulcus-based approach through anterior MFG and anterior IFG, advancing posteriorly under continuous DES until reaching reproducible speech arrest/morphosyntactic error sites at the posterior IFG boundary. Subcortical resection proceeded until stimulation at the posterior–superior depth disrupted speech initiation (functional stop) and until stimulation at the antero-inferior depth disrupted static visual–semantic processing (functional stop). Thus, the posterior IFG + posterior–superior subcortical boundary provides a mechanistic link to postoperative infinitive-only speech, whereas the inferior/deeper frontal floor boundary—where stimulation selectively impaired static recognition—offers a potentially direct intraoperative correlate for postoperative static-image agnosia with preserved dynamic recognition, consistent with partial disturbance of ventral semantic access pathways encountered at the inferior limit of the resection.

### 2.4. Postoperative Clinical Course

Immediately after surgery, the patient was alert and cooperative. Neurological examination showed no new motor or sensory deficits, and visual fields remained normal.

However, several neuropsychological changes were observed:

Speech production: Her spontaneous speech was markedly impoverished and primarily consisted of isolated infinitive verb forms (e.g., “to walk,” “to eat”), with reduced use of function words and grammatical inflections. This pattern resembled a dynamic aphasia with agrammatism, consistent with frontal involvement and disruption of frontal language networks.

Foreign accent syndrome and dysprosody: Subtle changes in prosody and accent were noted.

Language comprehension: Auditory comprehension for words and sentences remained largely intact.

Auditory and tactile naming: Naming based on auditory description and tactile exploration of real objects was relatively well-preserved.

Executive functions: Difficulties in planning and self-directed tasks persisted and were slightly accentuated in the immediate postoperative period.

### 2.5. Postoperative Visual Recognition Deficit

The most striking new deficit concerned recognition of static visual stimuli: The patient was unable to recognize static images of familiar objects and persons, including family photographs in an album and standardized pictures of common objects.

In contrast, she showed relatively well-preserved recognition of moving stimuli, such as characters and objects in movies or videos, and of real, three-dimensional objects manipulated in front of her (further details in Section 2.7).

Formal visual assessment included static image naming (she showed impaired naming of static line drawings and photographs despite preserved visual acuity and normal fields); object recognition (when presented with real objects, the patient was able to identify and name a higher proportion of items correctly compared to their corresponding static pictures); and static vs. moving stimuli (the same objects were first presented as static images (on paper or s screen) and then as moving objects (e.g., manipulated by the examiner) or dynamic video clips); recognition improved when objects were moving or presented in real space.

Letter and word reading were assessed, and reading accuracy and speed were within normal limits, indicating preserved orthographic processing and suggesting that the deficit was not a generalized visual identification or alexic disorder.

To further characterize the disorder, we examined drawing, copying, and matching tasks (matching pictures by category or function; picture–word matching) and analyzed error types (errors in visual naming were predominantly perseverative).

Overall, the pattern suggested an associative visual agnosia predominantly affecting static images: basic visual perception and copying were relatively well-preserved, semantic knowledge accessed through non-visual modalities was largely intact, but access to semantics from static visual input was disrupted. We refer to this clinically as a static visual agnosia, for which we use the descriptive label “astatopsia”.

### 2.6. Postoperative Imaging

Early postoperative MRI confirmed gross total resection of the frontal glioma (Figure 1). In addition, subtle signal changes were observed in the left occipitotemporal cortex, compatible with retraction-related injury or minor residual gliotic change.

The postoperative MRI showed likely gliotic changes/small contusion, to be confirmed in the long term. The lesion is probably not the only factor directly responsible for visual agnosia because there is a timing mismatch (symptoms are new and specific), and retraction injury is, here, present in a woman with insufficiently impaired basic vision to explain occipito-temporal failures. Long term imaging will clarify a hypothetic disruption of a distributed network involving occipitotemporal regions and their frontal connections.

### 2.7. Neuropsychological Assessment

A comprehensive battery of tests was administered before and after surgery, covering language, memory, attention, executive functions, visuospatial abilities, praxis, and visual object recognition.

Table 1 summarizes the main tests and gives a brief interpretation of each result.

This woman, with prominent anomia + dysexecutive syndrome (trouble planning/sequencing, keeping track of steps, initiating/completing everyday goal-directed tasks like sending emails or paying for parking), presented with a typical anomia pattern: low spontaneous naming, frequent “tip-of-the-tongue,” circumlocutions, improves with cueing (in a 60-item Boston Naming Test (BNT), she performed as spontaneously correct (38/60 and 52/60) with phonemic cues). Semantic knowledge was largely intact; naming problems were more about access/output (and executive control) than loss of meaning: the 52-items Pyramids and Palm Trees test (PPT) score was 49/52, showing correct but occasionally slow choices and a need for re-checking. Dysexecutive syndrome showed up as a disorganized copy strategy (piecemeal drawing, poor planning) and weak recall (encoding suffered because the copy was not organized): Rey–Osterrieth Complex Figure (ROCF) 36-point scoring revealed a score of 30/36, but it was qualitatively poor with a fragmented order and a slow (8 min), inefficient approach. She started the tasks (open email → search address → attach file → send; or park → open app → find location code → confirm payment) but failed to complete them reliably.

Two new, dissociated deficits emerged post-surgery: output restricted to infinitive verb forms and selective visual agnosia for static images (but dynamic recognition preserved). The overall condition was called “astatopsia.” In the picture-based tests, the patient did not reliably recognize the pictured item, and even when she did, she did not produce the required word form: the infinitive-only output prevented producing nouns even when the concept was accessed (responses drift to actions like “to cut,” “to drink,” “to write,” etc.). Post-surgery BNT dropped to 7/60 with a semantic cue and 8/60 with a phonemic cue. The qualitative response pattern included frequent action infinitives (“to pour,” “to sweep”) or vague infinitives (“to use”), plus “I don’t know” driven by static-image failures. If the examiner allowed non-standard scoring, e.g., credit for correct gesture/object use or written response, a higher “concept access” score was seen. Post-surgery PPT (46/52) remained relatively good (bypassing the static-picture recognition problem and not requiring spoken output), with a picture-low/word-high score split, suggesting “dissociation” as an effect of the selective static visual agnosia. ROCF (12/36) in the postoperative setting showed the patient to have difficulty recognizing static forms; the copy became severely impaired even if motor control was fine (poor copy → poor encoding → very poor recall), and copy quality was fragmented without a coherent global structure.

The neuropsychological results (pre- vs. post-surgery) are summarized in Table 1. An overview is also given by Schemes 1 and 2.

#### 2.7.1. Scheme 1 (Pre-Operative)

Main Preoperative Symptoms

- Anomia: Difficulty naming objects, despite fluent grammatical speech.
- Dysexecutive syndrome: difficulty with goal-directed behaviors such as sending emails and paying for parking, indicative of left dorsolateral prefrontal dysfunction.
- Motor, sensory, and visual fields intact.

Pattern summary:

BNT: impaired (spontaneous) with cue benefit → anomia with preserved knowledge;

PPT: near-normal → semantics mostly intact;

ROCF: copy score may look okay, but strategy is disorganized + recall poor → executive/organizational encoding problem.

Imaging (Figure 1)

- Preoperative MRI: Left inferior and middle frontal low-grade glioma.

#### 2.7.2. Scheme 2 (Postoperative)

Main Postoperative Findings

- Speech: limited to infinitive verbs only (e.g., “to walk,” “to eat”), lacking syntactic and morphological complexity.
- Visual Recognition: unable to recognize static images of common objects such as family album pictures but preserved recognition of moving/dynamic images such as movies.
- Auditory and tactile naming: preserved.
- Comprehension: intact.
- Executive functions: continued impairments in planning and self-directed tasks.
- Motor and sensory exam: no new deficits.
- Visual fields: normal.

Pattern summary:

BNT: collapses dramatically (picture input + noun output demands are both mismatched to the new deficits);

PPT: picture version drops, word version relatively well-preserved (classic dissociation since semantics are intact);

ROCF: copy and recall drop sharply due to impaired static-form processing plus worsened executive organization.

Imaging (Figure 1)

- Postoperative MRI: complete resection; subtle changes in left occipitotemporal cortex suggestive of retraction injury to be verified at long-term follow up.

All language tests, cognitive functions, attentional and menesic executive functions, praxic visuo-spatial skills, and gnosia were administered according to the evaluation protocol used in our center. Manual hemispheric dominance was strongly suggested by the Edinburgh test. In the postoperative period, the following were observed:

- Foreign accent syndrome;
- Dysprosody;
- Agrammatism with little use of functors (typical of broca aphasia) and prevalent communication based on the use of infinitive verbs;
- Static visual agnosia defined as “astatopsia”.

## 3. Discussion

This case illustrates a rare postoperative dissociation in a patient with left-hemisphere language dominance undergoing awake resection of a left frontal low-grade glioma: on the one hand, we note a dynamic aphasia-like language disorder, characterized by infinitive-only speech, reduced syntactic and morphological complexity, foreign accent syndrome, and dysprosody, pointing to disruption of the frontal language networks, including the inferior frontal and supplementary motor regions and their connections (e.g., frontal aslant tract—FAT) [12,13]; on the other hand, we note selective static visual agnosia, with impaired recognition of static images but relatively well-preserved recognition of moving stimuli and real objects in the context intact basic visual functions.

### 3.1. Characterization of the Visual Agnosia

The patient’s pattern is consistent with an associative visual agnosia: basic visual perception was relatively intact, as was semantic knowledge accessed via non-visual modalities, while recognition of static images of objects and persons was markedly impaired. Recognition improved when stimuli were presented as moving or as real objects in three dimensions. This dissociation between static image recognition and dynamic or object-based recognition aligns with prior reports of static visual agnosia and with the more general literature on dissociations between form and motion processing [14,15,16,17,18,19].

Our case shares some features with classic reports of static visual agnosia, such as the case described by Botez (1967) [8,15], in which patients showed selective difficulty with the recognition of static visual stimuli. It also differs from integrative visual agnosia, in which patients struggle to integrate elements of a visual scene into coherent wholes [5], and from Balint’s syndrome, which is typically characterized by simultanagnosia, optic ataxia, and ocular apraxia. In our patient, there was no evidence of optic ataxia or ocular apraxia, and no clear simultanagnosia was observed. Instead, the deficit appeared to be modality-specific (affecting static visual images) and predominantly associative. Unlike typical Balint’s syndrome, our patient did not show optic ataxia or ocular apraxia, and simultanagnosia was absent/mild. The dissociation between static image recognition impairment and the relatively well-preserved dynamic and object recognition differs in key aspects from the cases described by Botez and related integrative agnosias [14,15,16,17,18,19,20,21,22,23,24,25,26].

The authors propose the name “astatopsia” for the peculiar syndrome described in the paper, which, to the best of our knowledge, is not described in the literature. Our intention in coining “astatopsia” was not to replace established terminology but to provide a concise descriptive label for the specific pattern observed in this patient—namely, a prominent impairment in recognizing static images with relatively well-preserved recognition of moving stimuli and real objects. The term is Italian but comes from the Greek: A-(alpha privative)—stato (statós-stationary)—opsia (vision).

The coined term “astatopsia,” as used here, is intended as a descriptive label for this pattern of static image visual agnosia with relatively well-preserved dynamic and object recognition rather than as a proposal for a new nosological category. We explicitly situate this phenomenon within the broader class of static visual agnosias and dissociations between static and dynamic visual processing.

### 3.2. Anatomical Considerations: Frontal–Occipitotemporal Networks

The patient’s frontal tumor and its resection can account for the postoperative language and executive deficits. The dynamic aphasia-like pattern and agrammatism are compatible with disruption of the left inferior frontal gyrus and associated white matter tracts, including the FAT, which has been implicated in speech initiation, verbal fluency, and higher-order control [13]. Preoperative dysexecutive syndrome further supports involvement of the dorsolateral prefrontal cortex [3].

The emergence of static visual agnosia is more challenging to explain solely on the basis of frontal damage. Postoperative MRI showed subtle changes in the left occipitotemporal cortex, which is known to contribute critically to object recognition. Although these changes may reflect retraction-related injury rather than tumor infiltration, the postoperative MRI left occipitotemporal changes shows likely gliotic changes/small contusion to be confirmed at long-term follow up. We think the lesion is not responsible because there is a timing mismatch (symptoms are new and specific), and retraction injury is present, here, in a woman with insufficiently impaired basic vision to explain occipito-temporal failures. Long term imaging will clarify a hypothetic disruption of a distributed network involving occipitotemporal regions and their frontal connections. Moreover, the dual-stream model implicates occipitotemporal regions in ventral “what” processing and motion-sensitive areas (e.g., hMT/V5) in dorsal or extrastriate networks that can receive input via alternative geniculo-extrastriate pathways [3,4,12].

We therefore interpret the patient’s deficit as arising from a distributed disturbance of the ventral visual stream, potentially—but not certainly—including occipitotemporal regions and their connections to frontal language and executive areas. The relative preservation of motion perception and dynamic recognition is compatible with intact or partially spared dorsal/extrastriate pathways, similar to mechanisms proposed in blindsight and Riddoch-type phenomena [6,12]. The case thus supports the view that visual awareness and object recognition depend on coordinated interactions between occipitotemporal cortices, motion-sensitive extrastriate regions, and frontal networks involved in attention, semantic access, and report.

### 3.3. Frontal Contributions to Visual Awareness: Data vs. Speculation

Recent work suggests that frontal regions, including the prefrontal cortex and frontal eye fields, contribute to conscious visual perception and not merely to post-perceptual reporting [13]. Our patient’s combined frontal lesion and occipitotemporal changes are consistent with this network-based view, but the present single case does not allow for strong causal claims about frontal contributions to visual awareness.

We therefore distinguish between findings grounded in the data: the documented dissociation between static and dynamic/object-based recognition; preserved reading and non-visual–semantic access; and the presence of frontal language/executive deficits and subtle occipitotemporal changes.

More speculative hypotheses: We suggest that frontal areas may play a direct role in visual awareness by modulating access to semantic representations from visual input, possibly through long-range connections that include the FAT and other anterior–posterior tracts.

These speculative interpretations are offered as hypotheses consistent with current models, not as definitive conclusions.

### 3.4. Methodological Considerations and Limitations

This study has several limitations that must be acknowledged:

Language lateralization: Hemispheric dominance was inferred from the Edinburgh Handedness Inventory and Kimura’s dichotic listening test [8,9], which provide probabilistic evidence rather than definitive proof. More direct methods (e.g., fMRI language lateralization, Wada testing) were not employed.

Imaging: The occipitotemporal changes were subtle, and their exact relationship to specific white matter tracts could not be fully delineated. Future work with more detailed tractography and longitudinal imaging would help clarify structural–functional correlations: information regarding the integrity of the ILF and IFOF could be useful, particularly in light of the atypical clinical presentation [27].

Neuropsychological detail: The battery was tailored to the clinical constraints of awake neurosurgery. Some aspects of visual cognition (e.g., fine-grained measures of integrative agnosia or Balint-like symptoms) were not extensively tested preoperatively because there was no prior evidence of prosopagnosia, visuospatial neglect, or other visual attentional deficits.

Single-case generalisability: As a single case, the findings cannot by themselves establish a new syndrome. Instead, they illustrate a pattern that may be useful to recognize in future patients, particularly in the context of frontal neurosurgery.

Despite these limitations, this case provides a rare and documented example of selective static visual agnosia emerging alongside frontal language and executive deficits, highlighting the vulnerability of interconnected frontal–occipitotemporal networks.

In a right-handed patient with left-hemisphere language dominance suggested by standard non-invasive tests, awake resection of a left frontal low-grade glioma led to a dynamic aphasia-like speech disorder and a selective visual agnosia for static images, with relatively well-preserved recognition of moving stimuli and real objects. The clinical and imaging findings support the interpretation of an associative static visual agnosia (“astatopsia”) arising from disruption of a distributed network involving both frontal language/executive regions and occipitotemporal visual processing areas.

This case underscores the need for the following:

Comprehensive pre- and postoperative neuropsychological assessment, including detailed evaluation of visual recognition across static, dynamic, and object-based modalities.

Careful intraoperative mapping not only of language but also of higher-order visual and semantic functions, when feasible.

Consideration of white matter connectivity, including the frontal aslant tract and geniculo-extrastriate pathways, in neurosurgical planning and postoperative interpretation.

Future studies with larger case series and multimodal imaging will be needed to clarify how frontal–occipitotemporal networks support static and dynamic visual recognition and how their disruption leads to specific patterns of visual agnosia: the term “astatopsia” is used here solely as a descriptive shorthand and does not imply the definition of a new clinical syndrome; the observed deficit is best understood within the established framework of static visual agnosia and best interpreted within a distributed ventral stream network framework, rather than it being attributed to a single cortical locus.

## 4. Conclusions

In a right-handed patient with left hemisphere dominance, awake resection of a left frontal glioma led to infinitive-only speech and selective static image agnosia, highlighting the complex interplay between frontal language networks and ventral visual processing pathways. Preservation of motion perception supports the dual-stream model of visual cognition and emphasizes the need for careful intraoperative monitoring of both language and visual functions.

## Figures and Tables

**Table 1:** Before vs. after surgery tests pattern. BNT, Boston Naming Test; PPT, Pyramids and Palm Trees test; ROCF, Rey–Osterrieth Complex Figure.

TEST	Pre-op Score	Post-op Score	Description
**BNT**	52	8	collapses dramatically (picture input + noun output demands are both mismatched to the new deficits)
**PPT**	49	46	picture version drops, word version relatively well-preserved (classic dissociation since semantics are intact)
**ROCF**	30	12	copy and recall drop sharply due to impaired static-form processing plus worsened executive organization

Background color is typically used by our group to improve contrast and readability.

## Data Availability

The original data presented in this study are available onreasonable request from the corresponding author. The data are not publicly available due to privacy concerns.

## Abbreviations

The following abbreviations are used in this manuscript:

MRI	Magnetic resonance imaging
NAA	N-acetylaspartate
V1	Visual area 1
V5 or hMT	Middle temporal visual area (hMT or V5)
LGN	Lateral geniculate nucleus
FEF	Frontal eye field
FAT	Frontal aslant tract
ILF	Inferior longitudinal fasciculus
IFOF	Inferior fronto-occipital fasciculus
